# Clinical Study of Continuous Non-Invasive Blood Pressure Monitoring in Neonates

**DOI:** 10.3390/s23073690

**Published:** 2023-04-02

**Authors:** Anoop Rao, Fatima Eskandar-Afshari, Ya’el Weiner, Elle Billman, Alexandra McMillin, Noa Sella, Thomas Roxlo, Junjun Liu, Weyland Leong, Eric Helfenbein, Alan Walendowski, Arthur Muir, Alexandria Joseph, Archana Verma, Chandra Ramamoorthy, Anita Honkanen, Gabrielle Green, Keith Drake, Rathinaswamy B. Govindan, William Rhine, Xina Quan

**Affiliations:** 1 Department of Pediatrics, Division of Neonatal and Developmental Medicine, School of Medicine, Stanford University, Palo Alto, CA 94304, USA; 2 Division of Neonatology, LAC+USC Medical Center, Keck School of Medicine, University of Southern California, Los Angeles, CA 90033, USA; 3 Department of Emergency Medicine, Children’s Hospital Los Angeles, Los Angeles, CA 90027, USA; 4 Department of Anesthesia, School of Medicine, Stanford University, Palo Alto, CA 94304, USA; 5 PyrAmes Inc., Cupertino, CA 95014, USA; 6 Philips Healthcare, Sunnyvale, CA 94085, USA; 7 Children’s National Hospital, Washington, DC 20010, USA

**Keywords:** cNIBP, neonate, NICU, hypertension, hypotension, non-invasive blood pressure monitoring

## Abstract

The continuous monitoring of arterial blood pressure (BP) is vital for assessing and treating cardiovascular instability in a sick infant. Currently, invasive catheters are inserted into an artery to monitor critically-ill infants. Catheterization requires skill, is time consuming, prone to complications, and often painful. Herein, we report on the feasibility and accuracy of a non-invasive, wearable device that is easy to place and operate and continuously monitors BP without the need for external calibration. The device uses capacitive sensors to acquire pulse waveform measurements from the wrist and/or foot of preterm and term infants. Systolic, diastolic, and mean arterial pressures are inferred from the recorded pulse waveform data using algorithms trained using artificial neural network (ANN) techniques. The sensor-derived, continuous, non-invasive BP data were compared with corresponding invasive arterial line (IAL) data from 81 infants with a wide variety of pathologies to conclude that inferred BP values meet FDA-level accuracy requirements for these critically ill, yet normotensive term and preterm infants.

## 1. Introduction

Every year in the U.S., nearly half a million sick infants are hospitalized and undergo close monitoring of vital signs such as blood pressure (BP). In critically ill neonates, continuous BP monitoring is achieved by inserting a catheter into the lumen of an artery. This catheterization procedure is risky, invasive, and expensive—it requires skill and time, and infants may require sedation. Multiple attempts may be required to place the catheter due to small arteries [1,2,3]. A catheter may remain within the artery for several days and can lead to serious complications such as infection, bleeding, clots, tissue, and nerve damage [1,2,3,4].

Non-invasive alternatives to measure BP also have disadvantages. Oscillometry is the most widely used non-invasive method and measures BP by intermittently inflating a cuff. In general, continuous monitoring is preferred over intermittent, cuff-based, oscillometric monitoring for hypotensive neonates because premature neonatal BP values are often too low for an accurate measurement with a conventional cuff [5] and application of the cuff can perturb the patient to artificially elevate the measured BP values [6]. Moreover, the frequent use of cuffs can cause ischemic damage [7], particularly for patients with fragile skin such as premature neonates.

Other non-invasive techniques have been developed for the measurement of adult BP values, such as volume clamping using partially inflated finger cuffs, pulse transit time analysis using multiple sensors, and pulsewave analysis using cuffs or optical or radiative sensors [8]. These are summarized in Table 1. Almost all methods require calibration with a known BP measurement so that the measured features can be used to estimate BP [9,10,11], and none have been adopted for use with neonates. Machine learning (ML) methods can be used to analyze pulse waveform features and derive BP [12] and other hemodynamic parameters [13]. These include neural networks, probabilistic, decision tree, and rule-based induction.

Premature neonates have small wrists akin to an adult’s index finger and weigh as few as 400 g. For these infants, there is a critical need for a wearable, continuous, non-invasive BP (cNIBP) monitoring device that is accurate, affordable, and easy to use. For ease of use, it is necessary to overcome the need for calibration. Such a cNIBP device could eliminate the complications associated with IALs and obviate the need for sedation. Rapid and timely attachment could reduce latency for treating hypotension and potentially improve outcomes. Furthermore, less-skilled practitioners could use this device in resource-limited settings or during patient transport.

Here, we investigate the clinical feasibility and accuracy of estimating continuous BP in critically ill neonates with a wearable device (Boppli^TM^, PyrAmes Inc., Cupertino, CA, USA) using ML-based methods without the need for calibration.

## 2. Materials and Methods

### 2.1. Device Design and Working Principle

**Figure 1:**
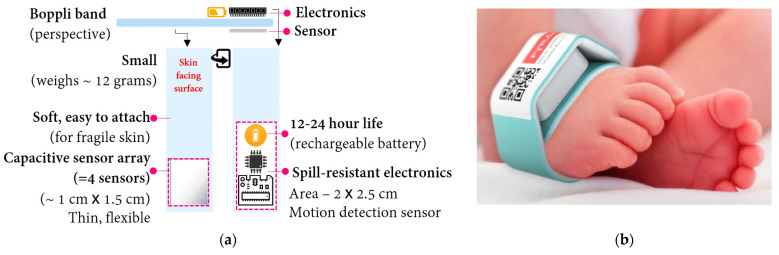
(**a**) Boppli sensor schematic: illustration of the sensor and wearable, continuous, non-invasive BP device for neonates (cross-sectional at the top; aerial view at the bottom); (**b**) Boppli sensor placement around the foot of an infant.

Boppli was designed to meet the BP monitoring requirements and physical dimensions of small, prematurely-born infants. It uses capacitive sensing methods, previously reported [8]. The thin (~50 μm), capacitance sensor array comprises four sensing elements. It is flexible and conforms to the contour of the limb. This extremely sensitive sensor is coupled with low-power electronics (Figure 1) to achieve a small form-factor and low weight (~12 g). The sensor and electronic components are incorporated into a disposable, foam band designed to ensure a snug fit without imposing excessive pressure on an infant’s delicate skin. The band does not use an adhesive, and its materials are hypoallergenic.

The device determines continuous BP in real-time by using two tandem processes: (1) capacitive sensing to quantify each pulse as a waveform, and (2) signal processing and machine learning methods to analyze and process the pulse waveform data to derive the systolic (SBP), diastolic (DBP), and mean arterial pressure (MAP).

#### 2.1.1. Capacitive Sensing

**Figure 2:**
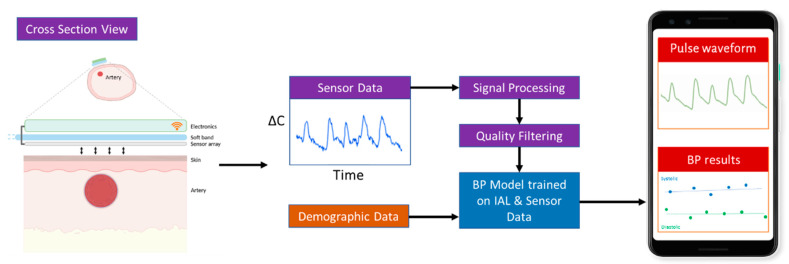
Overview of the working of the sensor and steps to infer blood pressure.

When the band is snugly wrapped around an infants’ wrist or foot, the sensor comes in contact with the skin that overlies the artery (Figure 2). In this configuration, the sensor and skin function like a capacitor. Each arterial pulse displaces the overlying skin towards the sensor. This displacement alters the capacitance (∆C) of the system. With every pulse, the capacitance will fluctuate. This fluctuation in capacitance is sensed by the electronics and is displayed as a waveform (“pulse-waveform”). These unscaled pulse-waveforms directly correlate with arterial BP and are wirelessly transmitted in real-time to a mobile device uniquely paired with each sensor. The capacitance data are collected at 125 Hz, and estimated BP values are updated at approximately 1 Hz.

#### 2.1.2. Pipeline for Real-Time Processing of Pulse Waveforms

The acquired pulse waveform data are streamed in real-time to a custom Android application (App). The App analyzes and displays the pulse waveforms (Figure 2). The measured pulse waveforms require additional signal processing and the application of machine learning trained algorithms to derive the systolic, diastolic, and mean arterial pressure (SBP, DBP, and MAP). These processing steps are summarized in Table 2.

#### 2.1.3. Data Quality

To extract robust parameters for further analysis, the pulse waveform data must have sufficient quality. Raw pulse waveform data from both the IAL and sensor can be noisy for multiple reasons such as active or passive infant movement.

To evaluate the usability of the measured pulse waveforms signal, a visual metric, *|Qv|*, was developed with a scale ranging from 0 (unusable) to 5 (best). This quality scale was based on a visual assessment of secondary peak resolution and the degree of signal noise by comparison to the corresponding IAL data according to a rubric. A training set of 10,000 pairs of Boppli/IAL pulse waveforms with *|Qv|* and regression coefficients was used to train a convolutional ANN (CNN) model to automatically grade Boppli and IAL pulse waveforms. The outputs of this model are a quality metric *|Q|* and estimated regression coefficient, *r*. The methods used to establish data quality are described in [8].

#### 2.1.4. Signal Processing

To decrease noise and increase signal quality i.e., signal-to-noise ratio (SNR), a Butterworth bandpass filter (order 2) with limits of 0.1 to 20 Hz was used to remove baseline shifts, respiratory effects, and high frequency noise. For infants on high-frequency oscillatory ventilation (HFOV), a notch filter based on the ventilation frequency was used to mitigate the parasitic noise due to subtle motion of the patient.

#### 2.1.5. Artifact Removal

Similarly, if the infant moves, the signal is momentarily distorted and that segment has low data quality *|Q|* and consequently cannot be used. Both IAL and sensor data were excluded if they did not meet the predefined *|Q|* and *r*. The IAL data were also required to have an SNR > 5 and to be within realistic ranges with a minimum value of 0 mmHg and maximum values of 200, 175, and 150 mmHg for SBP, MAP, and DBP, respectively. The pulse waveforms which met all these criteria were normalized and used to derive the SBP, MAP, and DBP with the BP model which was developed by using the process described below.

#### 2.1.6. BP Model

The purpose of the BP model is to recognize and correlate certain characteristics of the shape of a pulse waveform to BP values. Once learned, the BP model can automatically infer SBP, DBP, and MAP from the pulse waveforms.

Training Data: To enable learning, we developed an arterial line BP training/learning database by collating de-identified, historical IAL waveforms of infants and pediatric patients previously admitted at Lucile Packard Children’s Hospital Stanford (Stanford) and Children’s National Hospital and Boppli sensor data collected from patients with arterial lines in place at Stanford. These patient data were associated with patient age and weight but were not labeled with a diagnosis. The IAL and sensor data were processed to improve the signal quality and curated to remove artifacts as described above to make IAL data more suitable for learning [8].

Training database characteristics:

- A total of 306 infants under 5-years-old in the historical database and 95 patients collected in this study, no exclusions, both sexes.
- Up to 5000 pulse waveforms chosen randomly for each individual from a total of ~25,000 h of training data.
- Same quality preprocessing metrics as the data from the Boppli sensor.

When the IAL and sensor data were taken simultaneously, the two data streams for each patient were synchronized pulse-by-pulse by correlating the amplitudes of the pulse waveform signals and heart rates for a set of high-quality windows randomly distributed across the entire time series to obtain a global time lag value. A local time lag was then determined for each individual window to compensate for any missing data. A database was created from the synchronized windows where ground truth BP values were generated from the IAL data and associated with the sensor data for each window.

We used this database to train artificial neural networks to ‘learn’ features from pulse waveforms which could be correlated to SBP, DBP, and MAP values extracted from the peaks, valleys, and areas, respectively, of the IAL pulse waveforms. Finally, the ANN model was validated (Table 2, Step F) to ensure that it could perform well when presented with real-world data. Because of the limited amount of sensor data, 10-fold cross-validation (CV) sets were used (with 10% of the individuals with sensor data distributed into each CV set) where one set is held out as a test set while the nine other CV sets are used to train the model.

## 3. Clinical Study Design

The use of historical IAL data was approved by the Institutional Review Boards at Stanford University (IRB Protocol #47185) and at the Children’s National Hospital (IRB Protocol # Pro00014876). The observational study was approved by the Institutional Review Board at Stanford University (IRB Protocol #45892) and conducted in the neonatal (NICU) and cardiovascular intensive care units (CVICU) at Lucile Packard Children’s Hospital Stanford. The study complied with all relevant ethical guidelines for human subject research. Written consent was obtained from the parents of the study subjects prior to partaking in the study. We excluded those infants who were facing imminent demise and those undergoing extra corporeal membrane oxygenation (ECMO). After parental consent, we enrolled infants with umbilical arterial catheters (UACs) or peripheral arterial lines (e.g., radial, femoral, posterior tibial, axillary, or ulnar). The Boppli sensor was placed on an available wrist or foot with the sensor array overlying a pulse point. The wirelessly transmitted pulse waveforms were visualized in the App on a mobile device. The mobile device was kept in close proximity to the patient but not available to the clinical team. This was undertaken to ensure that clinical decisions were not made on the basis of the Boppli readings during the conduct of the clinical study.

### 3.1. Arterial-Line Data Collection

The catheter (UAC or peripheral arterial line) was connected to an electronic transducer for measuring continuous IAL BP. Measured BP waveforms sampled at 125 Hz were displayed on a Philips Intellivue (Philips Medical Systems, BG Eindhoven, The Netherlands) and stored in the Stanford data warehouse. The IAL data corresponding to each study subject was pre-processed as described above to improve quality and usability.

#### Correlation and Accuracy Metrics

Finally, we determined if the inferred, sensor-based BP (SBP, DBP, and MAP) correlated with BP from the IAL. Since the eventual clinical use of this device is to be determined by the US Food and Drug Administration (FDA), we compared the Boppli results to the accuracy specifications recommended by the FDA, i.e., a mean average error (MAE) < ±5 mmHg and standard deviation (SD) < 8 mmHg [14].

## 4. Results

The demographics for 81 infants enrolled in this study are shown in Table 3. These infants were admitted with a wide range of pathologies but were generally normotensive during the period of the study. Table 4 describes the characteristics of the invasive arterial line data. Table 5 provides characteristics of the IAL and Boppli placement locations. The gauge of the catheters used also varied (data not shown). Only patients with a weight below 5 kg and at least ten high-quality pulse waveforms were included in this analysis. 

### 4.1. cNIBP (Sensor)

The average length of data collection was eight hours per patient, with a minimum of 10 min and a maximum of 14.5 h. The total length of sensor data examined was ~750 h. The data recording was stopped when the arterial line was removed, when the battery ran out (~13–15 h), in anticipation of a procedure, or if the band was removed in error. In some cases, the band was removed or slipped to an incorrect position during extended periods of time, and only poor-quality data were collected during those periods.

### 4.2. Degree of Agreement

**Figure 3:**
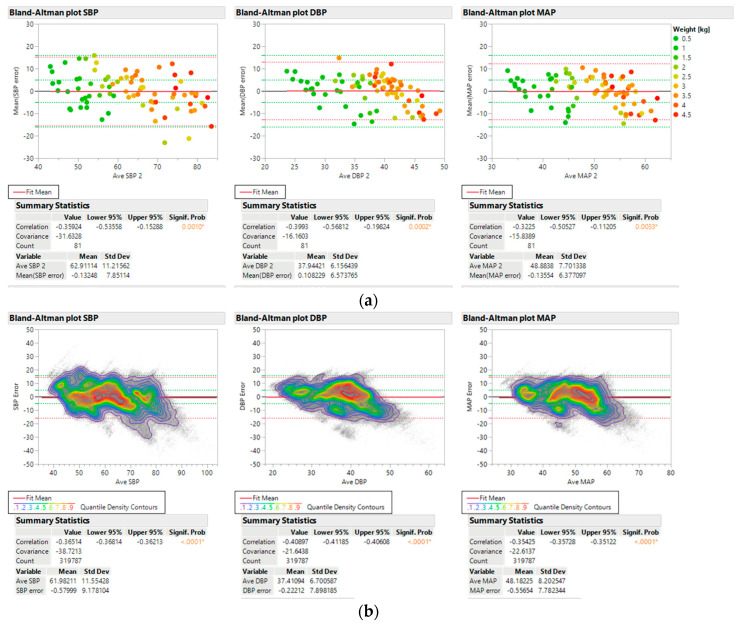
Bland–Altman plots for SBP, DBP, and MAP: (**a**) values averaged over each individual for 81 test patients; and (**b**) all data values without averaging. Bland–Altman plots represent a scatter plot of average versus difference of BP readings constructed for SBP, DBP, and MAP. Points are color coded by patient weight. Red dotted lines indicate 2 * SD of the calculated values. Green dotted lines indicate targets based on FDA guidelines for accuracy of MAE ≤ 5 mmHg and 2 * SD ≤ 2 * 8 mmHg. The figures were generated using JMP software version 16.2.0.

Bland–Altman analysis [15] for the mean difference between predicted BP (SBP, DBP, and MAP) and A-line BP demonstrates that SBP, DBP, and MAP predictions are within the FDA specifications when the sensor data *|Q|* > 2.5 and when averaged across each individual to account for the different number of data points per individual (Figure 3a). These analyses were performed in accordance with the guidelines for new BP devices provided by the American National Standards Institute/Association of the Advancement of Medical Instrumentation (AAMI)/International Organization of Standardization (Association for the Advancement of Medical Instrumentation and American National Standards Institute 2003). Predicted values were obtained from the pulse waveform data with the additional inputs of patient age and weight at the beginning of data collection. The points are color coded by patient weight and do not show a systematic trend of error with patient weight or BP values.

Bland–Altman plots with all derived data points without averaging are also shown with contour lines indicating point densities in Figure 3b. Again, the MAE specifications are met for SBP, DBP, and MAP and there is no systematic trend in error with BP values. However, all three BP values show increased variance and SBP does not meet the SD criterion.

**Figure 4:**
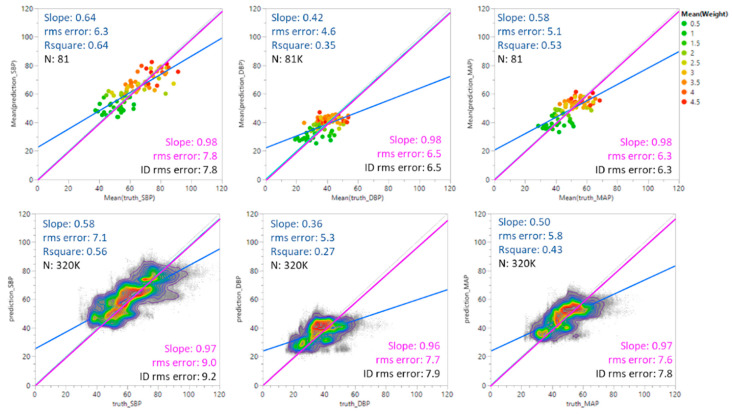
Predicted BP values vs. ground truth values. The blue line represents an unconstrained linear fit to the data. The green line is a linear fit constrained by a zero-intercept. The grey line is the identity (ID) fit where the predicted values equal the ground truth values. The figures were generated using JMP software version 16.2.0.

Figure 4 shows predicted SBP, DBP, and MAP values plotted against ground truth values. The top set of plots shows the results for average values weighted by individual; the bottom set shows all predicted values without averaging. Three linear fits are shown in each plot. The blue line shows the unconstrained fit to the data. The green line shows the fit of the data when the intercept is constrained to be 0. The grey line indicates the ideal (identity) case where predicted values equal the ground truth values with a slope of 1 and an intercept at 0. The root mean square (rms) errors are included for each fit for comparison.

Table 6 summarizes the degree of agreement for SBP, DBP, and MAP.

### 4.3. Effect of Gestational Age

Table 7 summarizes the MAE and SD values for the study population by gestational age at birth (GA) to understand if the BP algorithm is generalizable across the range of GAs found in NICU patients less than 5 kg in weight. The population was separated into three categories of GA: <28 weeks (extremely preterm, EPT), 28–37 weeks (moderately preterm, MPT), and ≥38 weeks (full-term, FT). These categories correspond to the extremes of gestational age as defined by the World Health Organization [1,4]. For MAP and DBP, all subsets meet the efficacy specification of MAE ≤ ±5 mmHg, SD ≤ 8 mmHg. SBP meets the MAE criterion for all subsets but the MPT subset does not meet the SD criterion.

The differences in accuracy between the GA groups were evaluated using the Tukey–Kramer analysis on JMP, a statistical package from SAS. This is a variant of the Tukey HSD method and uses SDs when there is an uneven number of data points per test period. Due to the range of data collection periods and variations in the quality of both IAL and Boppli data with time, the number of points varied by individual.

In the Tukey–Kramer (Tukey HSD) analysis, a letter code is assigned to each test group which can be used to gauge the statistical significance of differences between pairs of test groups. If the comparison of a pair of test groups did not exceed the threshold of significant difference, they were given the same letter code. If the analysis of their SDs exceeded this threshold, they were assigned different letter codes. A given test group may have multiple letter codes if it did not exceed the threshold of significant difference with several other test groups but some of the others differed significantly from one another and thus were given different letter codes. Pairs are considered significantly different if they do not share a letter code. The broader the difference between letter codes between a pair of test groups, the higher the likelihood that the pairs are significantly different. In Table 7, all groups show the same letter code and thus there does not appear to be a significant difference between GA groups.

**Figure 5:**
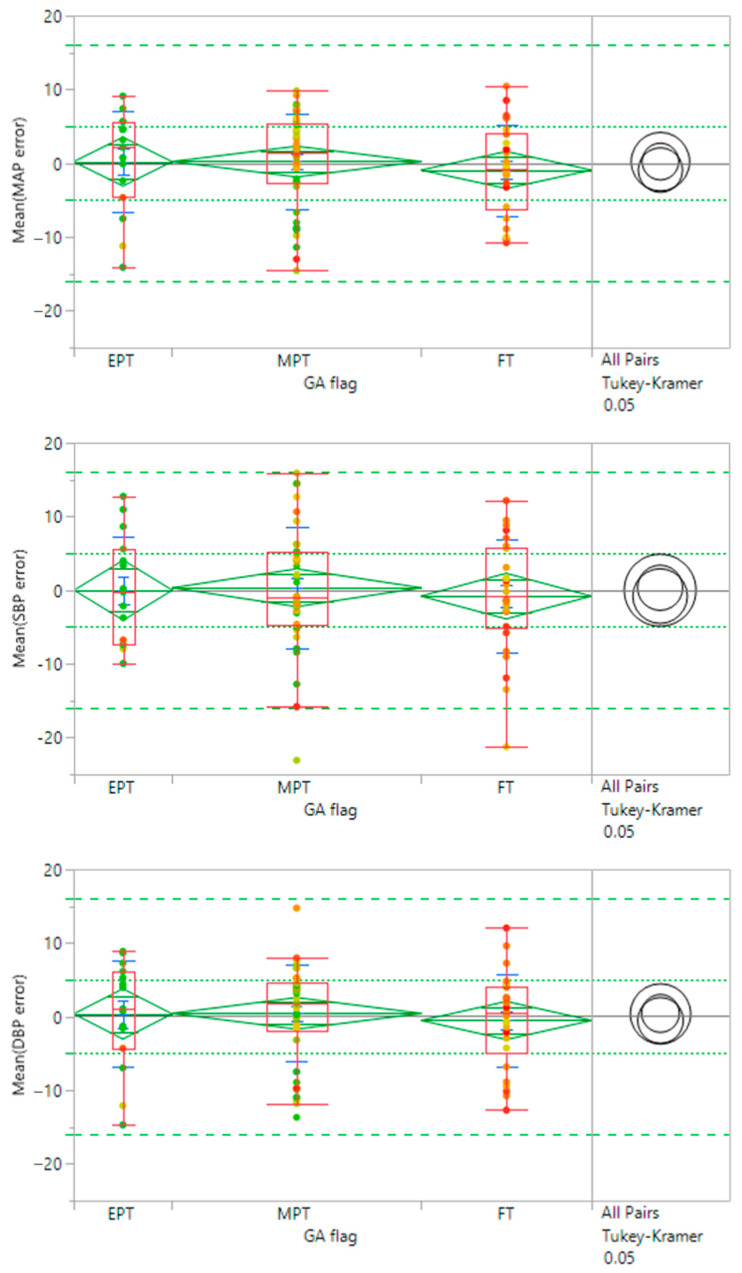
Effect of gestational age (GA) on efficacy. Groups categorized by prematurity are compared through mean diamonds (green) and Tukey–Kramer circles (black). EPT (<28 wks GA), MPT (28–37 wks GA), FT (≥38 wks GA). Overlap between diamonds and circles indicates there is no significant difference between groups. Mean diamonds are contained within the FDA guidelines for accuracy (dotted green lines). Targets for 95% confidence levels are indicated with dashed green lines. Points are color-coded by patient weight.

Figure 5 shows the comparison results for the three GA categories of infants. Data points are colored by patient weight at the time of the study. A good overlap between the mean diamonds (green) and the Tukey–Kramer circles (black) indicates there is no significant difference between the GA groups. All subsets show MAP, SBP, and DBP mean values within the specification for accuracy (green dotted lines at ±5 mmHg error) and the 95% confidence level (green dashed lines at ±16 mmHg). As such, there does not appear to be an impact of gestational age on accuracy. Similar analyses show no significant trends in efficacy due to age, weight, race/ethnicity, IAL location, or Boppli location.

**Figure 6:**
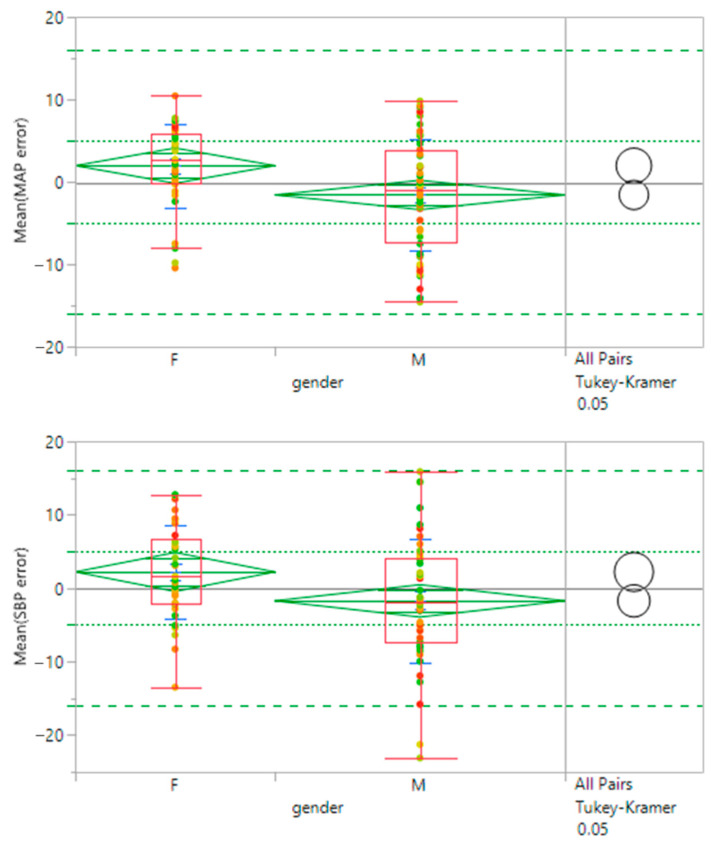

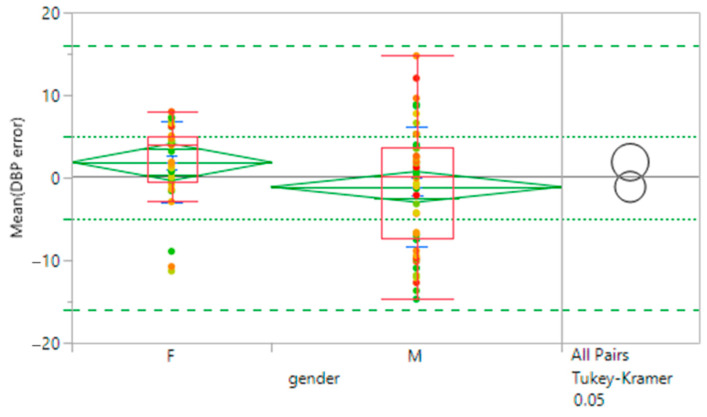
Effect of sex on efficacy. Female and male patient subsets are compared through mean diamonds (green) and Tukey–Kramer circles (black). Minimal overlap between diamonds and circles indicates there is a significant difference between the two groups. Mean diamonds are contained within the FDA guidelines for accuracy (dotted green lines). Targets for 95% confidence levels are indicated with dashed green lines. Points are color-coded by patient weight.

There is some indication of a significant difference due to sex although the results for both male and female cohorts meet the accuracy (MAE) guidelines. There is more variance for the male subset which exceeds the target of SD ≤ 8 mmHg for SBP. The results are shown in Table 8 and Figure 6.

## 5. Discussion

A non-invasive approach to monitoring BP is a vital unmet need in neonatal critical care. Currently, there are no commercially available neonatal cNIBP devices. Solving this unmet need can provide better, safer BP monitoring for the ~400,000 NICU patients annually in the U.S. [16] and avoid the pain, stress, and risks of IALs. The novel Boppli cNIBP device can continuously and non-invasively infer BP without the need for external calibration. This is significant because it reduces the nursing burden at the bedside. The device does not use adhesives, which is relevant for premature neonates who lack a protective layer in the skin, i.e., stratum corneum [17]. It is less perturbing to the patient than an IAL, cuff, or other bulky non-invasive options. This device can be strapped on like a wrist-watch and can be used while transporting patients. The electronics can be reused to a lower cost of care. Consequently, this device can change the management of neonates by avoiding arterial line placement in neonates who strictly need monitoring for BP issues.

A few prior studies have suggested that non-invasive methods of BP overestimate invasive arterial lines [18,19]. These studies used intermittent, cuff-based methods unlike the continuous, non-cuff-based method described here. Other studies have shown good agreement for non-invasive methods of BP measurement [5,20]. We expect that this device, once cleared by the FDA and adopted for clinical use, will potentially eliminate the complications associated with invasive arterial lines. This transition will pave the way for enabling continuous non-invasive monitoring, early detection of changes in BP, timely intervention, and tracking of the response to treatment. Although this study focuses on neonates, the technology platform is applicable to all age groups, and limited only by the availability of arterial waveform data for building neural network models.

The study had limitations. All enrolled neonates were critically ill but were normotensive. We acknowledge that the utility of this device and its accuracy is of utmost value in hypotensive patients and such a study is intended in the future. The correlation between a gold-standard arterial line and cNIBP sensor can depend on the precise location of the arterial line. We have not accounted for this issue in the current study. Critically ill infants often have fluids running through their umbilical lines. This can dampen the observed and recorded signals. Comparing the cNIBP signal from a limb to a presumably corrupted waveform, deemed as a “gold-standard” can be inaccurate. Another limitation is due to the blinding of the data to the clinical team which led to periods of time where the data quality was insufficient for analysis. In future studies, we will display the pulsewave to the clinical team to provide a visible cue of data quality. We have observed that the fraction of data with usable quality increases when the clinicians are able to see the Boppli signal in real time.

Our study also has strengths. First, we demonstrated the accuracy of the device in estimating BP in real-world ICU settings and in infants with a wide variety of clinical conditions. Additionally, capturing unscaled data is innovative because it overcomes the need to calibrate for skin type, edema, patient size and shape, sensor positioning, and temperature sensitivity. The BP model was generated with data obtained from 306 patients. We believe that additional data can refine the algorithms and increase accuracy. In several instances, IAL data were distorted due to over/underdamping and artifacts from transducer flushing, kinking of catheters, movement artifacts, BP cuff inflations, and high frequency oscillatory ventilation. However, using filtering techniques, we have extracted underlying cNIBP waveforms. This work is novel and significant because it can impact clinical practice and simultaneously enable real-time analytics [21].

## 6. Conclusions

This study is significant because it addresses a critical gap faced by clinicians treating infants in the neonatal period. We have investigated the accuracy of a novel wearable device that utilizes capacitive sensing and algorithms trained through machine learning in tandem to infer BP, continuously and non-invasively, without external calibration. We showed that the use of the device is feasible for critically ill term and preterm infants with a wide variety of pathologies—from serious cardiac ailments, pre- and post-operative cases, neurologic conditions, and prematurity. The ANN model used in this study is one of several models we have developed to estimate BP in real-time without the need for calibration. The wearable meets the FDA specifications for accuracy and is a practical choice in clinical settings—it is easy to attach and operate and provides continuous BP readings with no latency. A pivotal multi-center study to assess accuracy and submit data to the FDA for device clearance is underway.

## Figures and Tables

**Table 1:** Methods for determining BP. Adapted * from Ref. [8].

	Method	Measurement	Example Companies	FDA
Cuff	Finger, tabletop	Continuous	BMEYE, Finapres, ADI, Biopac, Edwards (ClearSight), CNAP	Yes
	Finger, wearable	Continuous	Caretaker	Yes
	Wrist	Intermittent	Omron, H2Care	Yes
Cuffless	PPG	Continuous	Aktiia, BioBeat, Apple, ASUS, Samsung, Sensifree	Yes
	PWV, PTT	Continuous	Vital Insight, Quanttus, Scanadu, Blumio, Sibel	No
	Tonometer	Continuous	Tensys, HealthStat, LiveMetric	Yes
	Capacitance	Continuous	PyrAmes, Vena Vitals	Submitted

**Abbreviations**: **PWV**: Pulse wave velocity; **PTT**: Pulse transit time; **PPG**: Photoplethysmography.

**Table 2:** Pipeline for real-time processing of pulse waveforms and artificial neural network (ANN) training.

Modules	Steps	Relevance
**A. Quality Model**	**Select** data **Exclude** data segments **Quality ranking** **Classification elements**	Boppli Band has an array of four sensors. The algorithm chooses data from the best sensor. Infant moves ⇨ pulse waveform excluded if quality value is below the threshold. Automated PW quality rating (0—bad, 5—good) using ANN, correlation coefficient Boppli/IAL. Algorithm automatically *detects* if the pulse waveform is corrupted by HFOV.
**B. Signal Processing**	**Noise filtering/** **↑SNR**	If Classification element detects HFOV ⇨ then notch filter. Normalize pulse waveform.
**C. BP Model**	**CNN trained** on pulse waveform and IAL **SHAPE**	Determines systolic, diastolic, mean arterial blood pressure.
**D. Training Data**	**Obtain Boppli/IAL data**	Sources: Stanford and CNH patient data warehouse; Stanford Boppli/IAL data collection.
**E. Data Curation**	**Clean** IAL data **Synchronize** data	Removes artifacts due to motion, damping, and other IAL operational issues. Synchronize Boppli and IAL data taken simultaneously.
**F. Training and Testing**	**Cross-validation (k-fold)** **Model Ranking**	Inputs: pulse waveform data, age, and weight. Splits data into 10 groups. Takes one group as a test and the remainder as training. Recursively tests model using TensorFlow and proprietary code. Choose best model that minimizes MAE and SD while optimizing slope and correlation coefficient of regression fit of estimated vs. ground truth values.

**Abbreviations**: **PW**: Pulsewave; **ANN**: artificial neural network; **HFOV**: high frequency oscillatory ventilation; **MAE**: mean average error; **SD**: standard deviation; **IAL**: intraarterial line; **CNH**: Children’s National Hospital.

**Table 3:** Characteristics of the study cohort. Normotensive, not on any inotropic support during the measurement period.

Study Cohort	Patient Characteristics (N = 81)		
**Age (days)**	Minimum Maximum Average Median	1 150 17 4	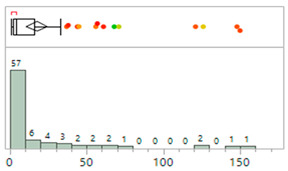
**Gestational age (weeks)**	Minimum Maximum Average Median	24.14 41.29 34.33 37.00	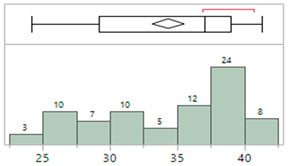
**Weight (kg)**	Minimum Maximum Average Median	**0.55** **4.85** **2.60** **2.80**	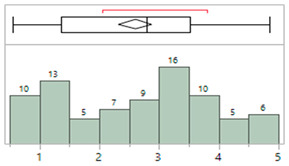
**Sex, n (%)**	60.5% male, 39.5% female		
**Race/Ethnicity**	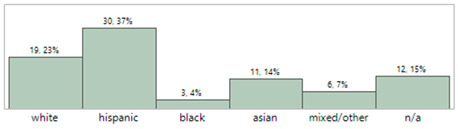		
**Primary diagnosis at time of measurement**	Cardiac (34), gastrointestinal (3), hyperbilirubinemia (2), multisystem congenital (4), neurological (8), prematurity (21), respiratory (4), Trisomy 21 (1), combination issues (3), and pulmonary hypertension (1) issues		

**Table 4:** Characteristics of the invasive arterial line data.

**IAL MAP (mmHg)** **individual means**	Minimum Maximum Average Median	29 68 49 50	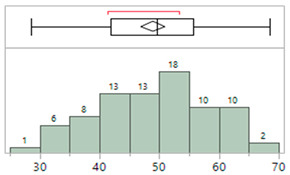
**IAL SBP (mmHg)** **individual means**	Minimum Maximum Average Median	38 91 63 61	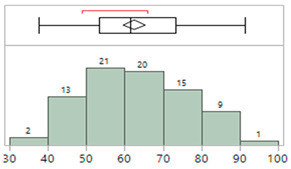
**IAL DBP (mmHg)** **individual means**	Minimum Maximum Average Median	19 54 38 38	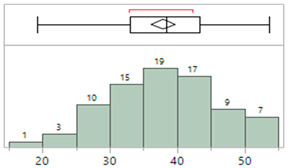

**Table 5:** Characteristics of the IAL and Boppli placement locations.

**IAL location(s)** (Some patients had more than one IAL)	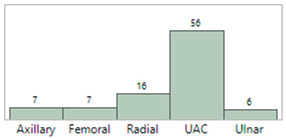
**Boppli location(s)** (Some patients wore more than one Boppli)	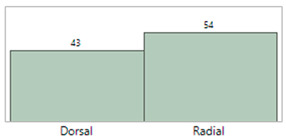

**Table 6:** Summary of degree of agreement for SBP, DBP, and MAP including agreement with FDA guidelines (green) and agreement falling outside FDA guidelines (red).

	Systolic BP		Diastolic BP		Mean Arterial BP		FDA Guidelines	
	MAE	SD	MAE	SD	MAE	SD	MAE	SD
**Individual averages** **(N = 81)**	−0.1	7.9	0.1	6.6	−0.1	6.4	≤±5 mmHg	<8 mmHg
**All points (N = 327 K)**	−0.6	9.2	−0.4	7.9	−0.6	7.8		
	***r^2^***	**Slope**	***r^2^***	**Slope**	***r^2^***	**Slope**		
**Individual averages** **(N = 81)**	0.64	0.64	0.35	0.42	0.53	0.58		
**All points (N = 327 K)**	0.56	0.58	0.27	0.36	0.43	0.50		

**Table 7:** Efficacy by gestational age.

GA	N	MAP			SBP			DBP		
		MAE	SD	Letter Code *	MAE	SD	Letter Code *	MAE	SD	Letter Code *
EPT: <28 wk	15	0.2	6.9	A	−0.0	7.3	A	0.4	7.3	A
MPT: 28–37 wk	38	0.2	6.4	A	0.3	8.3	A	0.4	6.6	A
FT: ≥38 wk	26	−0.9	6.2	A	−0.8	7.8	A	−0.5	6.4	A

* Letter codes not connected by the same letter are significantly different.

**Table 8:** Efficacy by sex.

	N	MAP			SBP			DBP		
		MAE	SD	Letter Code *	MAE	SD	Letter Code *	MAE	SD	Letter Code *
Female	33	2.0	5.1	A	2.2	6.4	A	1.9	4.9	A
Male	48	−1.6	6.8	B	−1.7	8.4	B	−1.1	7.3	B

* Letter codes not connected by the same letter are significantly different.

## Data Availability

Data presented in this document that were generated solely by PyrAmes are available from the corresponding author upon reasonable request. The data are not publicly available due to sensitive personal data that were collected during clinical studies on patients. Data generated or provided by Stanford University and Children’s National Hospital are restricted by the terms of contractual agreements and are available upon reasonable request with the permission of those organizations and if the requestor agrees to comply with the European General Data Protection Regulation and not to attempt to trace the origin of the data.

## Code Availability Statement

The custom code used in this study is copyrighted by PyrAmes Inc. Any commercial use including the distribution, sale, lease, license or other transfer of the code to a third party is prohibited. For inquiries regarding commercial use of the code, please contact xquan@pyrameshealth.com.
